# Draft Genome Sequence of *Micromonospora* sp*.* Strain HK10, Isolated from Kaziranga National Park, India

**DOI:** 10.1128/genomeA.00559-15

**Published:** 2016-08-11

**Authors:** Madhumita Talukdar, Dhrubajyoti Das, Chiranjeeta Borah, Hari Prasanna Deka Boruah, Tarun Chandra Bora, Anil Kumar Singh

**Affiliations:** Department of Biotechnology, CSIR-North East Institute of Science and Technology, Jorhat, Assam, India

## Abstract

We report the 6.92-Mbp genome sequence of *Micromonospora* sp. HK10, isolated from soil samples collected from Kaziranga National Park, Assam, India. The full genome of strain *Micromonospora* sp*.* strain HK10 consists of 6,911,179 bp with 73.39% GC content, 6,196 protein-coding genes, and 86 RNAs.

## GENOME ANNOUNCEMENT

Members of the *Micromonospora* genus are widely distributed in terrestrial, aquatic, and marine environments (1–4), but few of them have been isolated from nitrogen-fixing root nodules (5–7). *Micromonospora* species are well known for synthesizing antibiotics, especially aminoglycoside, enediyne, and oligosaccharide antibiotics. Despite the production of various useful antibiotics by *Micromonospora* species, the full genomes of only six strains have been sequenced to date. To enhance the knowledge base of *Micromonospora*, we contribute here a new genome sequence of *Micromonospora* sp. strain HK10, which was isolated from a soil sample of Kaziranga National Park, Assam, India.

The *Micromonospora* sp. HK10 genome was sequenced with the Illumina NextSeq 500 format using a paired-end 2 × 150-bp library. The raw data were trimmed using Trimmomatic version 0.30 (8) for high-quality read length (cutoff quality score, 20). A total of 8,896,194 high-quality vector-filtered reads were used for assembly with CLC Genomics Workbench version 6 (CLC bio, Denmark). The final assembly contains 294 contigs with a total size of 6,911,179 bp, a GC content of 73.39%, and an *N*_50_ contig length of 61,860 bp; the longest contig assembled measures 352,445 bp. The draft genome of *Micromonospora* sp. HK10 was annotated for protein-coding genes with the help of Prodigal version 2.60 (9), and a total of 6,282 CDSs were predicted.

All of the CDSs were further analyzed using BLASTx and the Kyoto Encyclopedia of Genes and Genomes (KEGG). BLASTx analysis was performed for predicted protein-coding genes using the nonredundant protein database (*E* value = 10^−5^); as a result, 6,282 CDSs were observed and annotated, including 3 rRNAs and 58 tRNAs. The assignment of orthologous genes and the mapping of the CDSs to the biological pathways were performed using the KEGG server; a total of 1,052 genes were annotated. The gene ontology mapping was performed using Blast2GO version 2.0 (10), and the overall gene ontology distribution was 43.1% for molecular function, 43.4% for biological process, and 13.5% for cellular function. A maximum number of 2,086 out of 6,282 CDSs were matched with orthologs in *Micromonospora* sp. L5 (5), which is the closest member to HK10; the second closest is *Micromonospora aurantiaca* ATCC 27029 (NC_014391) with 1,688 ortholog matches.

Our annotation results indicated that HK10 has genes for carbohydrate metabolism (tricarboxylic acid cycle, pentose phosphate pathway, glycolysis, and glyconeogesis), energy metabolism (oxidative phosphorylation), lipid metabolism (fatty acid biosynthesis and degradation), amino acid metabolism, glycan metabolism (peptidoglycan, lipopolysaccharide), and cofactor and vitamin metabolism. Various pigments (porphyrin, carotenoid), antibiotic coding clusters (ansamycins, tetracycline, vancomycin group of antibiotics, neomycin, novobiocin, penicillin, and cephalosporin), and polyketide synthase coding genes have been annotated. Several genes have also been annotated related to xenobiotic biodegradation and their metabolism (benzoate, chloroalkane, toluene, xylene, naphathalene, polycyclic aromatic hydrocarbon, etc).

### Nucleotide sequence accession number.

This whole-genome shotgun project has been deposited at DDBJ/EMBL/GenBank under the accession number JTGL00000000. The version described in this paper is the first version.
